# Correlation Between Outcomes of Surgical Correction of Atlantoaxial Instability in Small Dogs and Magnetic Resonance Imaging Abnormalities

**DOI:** 10.3390/ani16091347

**Published:** 2026-04-28

**Authors:** Chompoonuch Panya-ud, Woranika Bunkrasin, Kanokwan Keadwut, Pakthorn Lewchalermwong, Wutthiwong Theerapan, Waraporn Aumarm

**Affiliations:** 1 Kasetsart University Veterinary Teaching Hospital, Bangkhen Campus, Faculty of Veterinary Medicine, Kasetsart University, Bangkok 10900, Thailand; chompoonuch.pa@ku.th (C.P.-u.); woranika.bu@ku.th (W.B.); kanokwan.ke@ku.th (K.K.); pakthorn.l@ku.th (P.L.); 2 Department of Companion Animal Clinical Sciences, Faculty of Veterinary Medicine, Kasetsart University, Bangkok 10900, Thailand; wutthiwong.t@ku.th

**Keywords:** atlantoaxial instability, AAI, magnetic resonance imaging, dogs, small breed, surgical outcome

## Abstract

Congenital atlantoaxial instability (AAI) occurs more often in young, small-breed dogs, usually resulting from malformations in the craniocervical junction. These malformations cause atlantoaxial subluxation or luxation and compression of the cervical spinal cord. As a result, dogs can have varying levels of neurological problems. While surgery is commonly used to realign the vertebrae, outcomes after surgery vary widely, and complications are common. Magnetic resonance imaging (MRI) helps identify abnormalities in the vertebral canal, spinal cord damage, and other brain diseases, which can affect the dog’s prognosis. This retrospective study assessed MRI findings and their association with surgical outcomes in 20 dogs with AAI. Older age and certain MRI results—such as severe ventral spinal cord compression associated with greater preoperative neurological severity and syringomyelia—were associated with less favorable surgical outcomes. Many MRI abnormalities were found, but not all predicted poor outcomes. These results show the importance of thorough MRI before surgery and also suggest that a younger age and not having syringomyelia are good indicators of improved recovery after AAI surgery.

## Simple Summary

Atlantoaxial instability (AAI) is a condition affecting the cervical vertebrae that commonly causes spinal cord compression and neurological issues in small-breed dogs. While surgery is the standard treatment for stabilizing joints, predicting which dogs will recover well remains a challenge. This study examined the relationship between pre-surgical magnetic resonance imaging (MRI) findings and long-term recovery in 20 dogs. Our results show that older dogs and those with specific MRI abnormalities—particularly severe spinal cord compression and syringomyelia—tend to have more severe neurological signs before surgery. However, only syringomyelia was associated with a slower or less complete postoperative recovery. These findings highlight that a detailed MRI scan prior to surgery is essential for veterinarians to provide an accurate prognosis and help owners understand their pets’ likely outcomes.

## 1. Introduction

Congenital atlantoaxial joint instability is overrepresented in young, small dog breeds, including the Yorkshire Terrier, Toy Poodle, Pomeranian, Chihuahua, and Pekingese [1,2,3]. The disorders resulting from congenital malformations affect the region extending from the occipital bone to the craniocervical vertebrae (craniocervical junction abnormality), including occipitoatlantoaxial malformation, dens abnormalities (including hypoplasia, aplasia, or abnormal angulation), and atlantooccipital overlapping. Instability of the atlantoaxial joint results in luxation or subluxation, leading to compression and contusion of the cervical spinal cord, which causes varying degrees of neurological dysfunction [2,4,5,6,7]. Surgical treatment is indicated for subluxation reduction and joint stabilization, particularly in patients with neurological deficits or neck pain unresponsive to medical treatment [7,8,9].

The diagnosis of atlantoaxial subluxation is performed using plain radiographs, computed tomography (CT), or magnetic resonance imaging (MRI). Cervical vertebral column plain radiographs may detect dorsal displacement of the axis into the vertebral canal, an increased angulation between the dorsal laminae of C1 and C2, an increased distance between the arch of C1 and the spinous process of C2, and hypoplasia, aplasia, or dorsal angulation of the dens [3,7]. While lateral radiography of the C1–C2 joint during neck flexion may confirm instability, it can worsen neurological signs [3,7,10]. Although CT scans provide detail of bone structures and fractures and aid in the development of three-dimensional reconstructions for surgical planning, they do not provide information about soft tissue structure, craniocervical junction, or spinal cord—including information on the ligamentous structure and the presence of hemorrhage, syringomyelia, and abnormal intramedullary intensity—and cannot identify concurrent neurological disease, which may be associated with the prognostic results that MR images can provide [2,7,11]. Clinical outcomes in dogs undergoing surgery for atlantoaxial instability (AAI) are highly variable, and the condition is associated with a relatively high rate of postoperative complications [8,12,13]. These uncertainties can complicate clinical decision-making for both veterinarians and pet owners. Magnetic resonance imaging (MRI) of the vertebral column provides essential information for diagnosis, the evaluation of spinal cord damage, and the detection of concurrent diseases. However, in the veterinary literature, only a limited number of studies have described MRI findings and craniocervical junction abnormalities in dogs with atlantoaxial instability [2,11,14,15,16]. Furthermore, few studies have investigated whether the severity and type of MRI abnormalities correlate with postoperative neurological improvement following surgical stabilization.

In this study, our aims were to describe MRI characteristics in dogs with atlantoaxial subluxation and to evaluate the relationship between MRI abnormalities and surgical outcomes by comparing preoperative and postoperative neurological grades in small-breed dogs.

## 2. Materials and Methods

### 2.1. Case Selection

A retrospective review of surgical records of atlantoaxial instability in dogs at Kasetsart University Veterinary Teaching Hospital, Thailand, from January 2017 to December 2025 was conducted. Inclusion criteria for the study required a complete clinical history, neurological examination (pre- and postoperative), and preoperative magnetic resonance imaging, with no age, sex, or breed restrictions. Dogs diagnosed with atlantoaxial instability were graded on a 0–5 scale according to a clinical neurological grading system, as follows: grade 0, neurologically normal; grade 1, spinal hyperesthesia without neurological deficits; grade 2, ambulatory para- or tetraparesis; grade 3, non-ambulatory para- or tetraparesis; grade 4, para- or tetraplegia with intact deep pain perception; and grade 5, para- or tetraplegia with loss of deep pain perception [9,17,18].

Dogs without postoperative follow-up, those that died postoperatively, or dogs with musculoskeletal conditions that interfered with accurate neurological evaluation were excluded from the study.

### 2.2. Data Collection

#### 2.2.1. Preoperative Imaging

The diagnosis of atlantoaxial instability was confirmed through magnetic resonance imaging (MRI) using a 1.5-Tesla scanner (Siemens Magnetom Essenza, Erlangen, Germany). MRI was performed under general anesthesia induced with intravenous propofol (4–6 mg/kg), with dogs positioned in dorsal recumbency. All dogs underwent MRI examination, including T1- and T2-weighted sagittal and transverse images of the craniocervical junction. Post-contrast T1-weighted images with fat suppression were obtained following the intravenous administration of a paramagnetic contrast medium (gadoteridol, 0.5 mmol/mL) at a dose of 0.1 mmol/kg. All MRI studies were reviewed by a board-certified veterinary radiologist.

Cases were classified by head position during diagnostic imaging as extended (angle < 25°) or flexed (angle ≥ 25°). The angle of the head position measures the intersection of two lines, including one line drawn from the tuberculum sellae to the base and the other extending from the craniodorsal to the caudodorsal margin of the vertebral body of the axis [14]. The abnormal characteristics of the MR image were assessed, including lateral ventricular enlargement, dorsal compression of the atlantoaxial band, cerebellar compression, spinal cord compression, ventral compression index, presence of syringomyelia, and Chiari-like malformation [6]. Assessment of atlantooccipital overlapping (AOO) and other craniocervical junction abnormalities was not systematically performed in this cohort.

#### 2.2.2. MRI

##### Lateral Ventricular Enlargement

**Figure 1:**
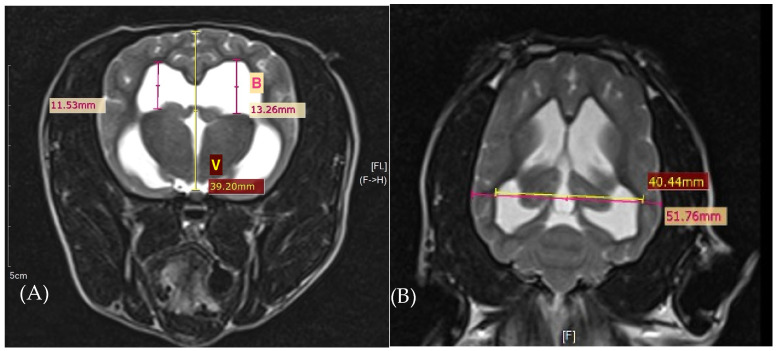
Method for measurement of lateral ventricle enlargement with the (**A**) ventricle/brain ratio in the transverse MR image and the (**B**) ventricle/brain ratio in the dorsal MR image (Laubner et al., 2015 [21]).

Lateral ventricular enlargement was defined as a ventricle-to-brain (V/B) ratio exceeding 15% on transverse MR images, as previously described (Vullo et al., 1997) [19]. The V/B ratio was calculated by dividing the height of the lateral ventricle (V) by the distance from the dorsal ventricular surface to the pituitary gland (B) [2,20] (Figure 1).

Dogs with clinically significant hydrocephalus had a ventricle/brain index greater than 0.6, which can differentiate internal hydrocephalus from ventriculomegaly. The ventricle/brain index is calculated by dividing the greatest continuous distance between the inner edges of the lateral ventricles by the maximum width of the brain parenchyma in the same dorsal T2 image [21].

In cases of asymmetric lateral ventricles, the asymmetry ratio was used for classification as follows: mild (1–2:1), moderate (2–3:1), and severe (>3:1) [22].

##### Dorsal Compression of the Atlantoaxial Band/Dural Fibrous Band

The atlantoaxial band, located at the atlantoaxial junction, is associated with Chiari-like malformations and other craniocervical malformations, and clinical improvement has been observed after surgical removal. These bands are visible as hypointense, marginated tissue at the dorsal atlantoaxial junction in T2W MR images, causing spinal cord compression [23,24].

##### Spinal Cord Compression

**Figure 2:**
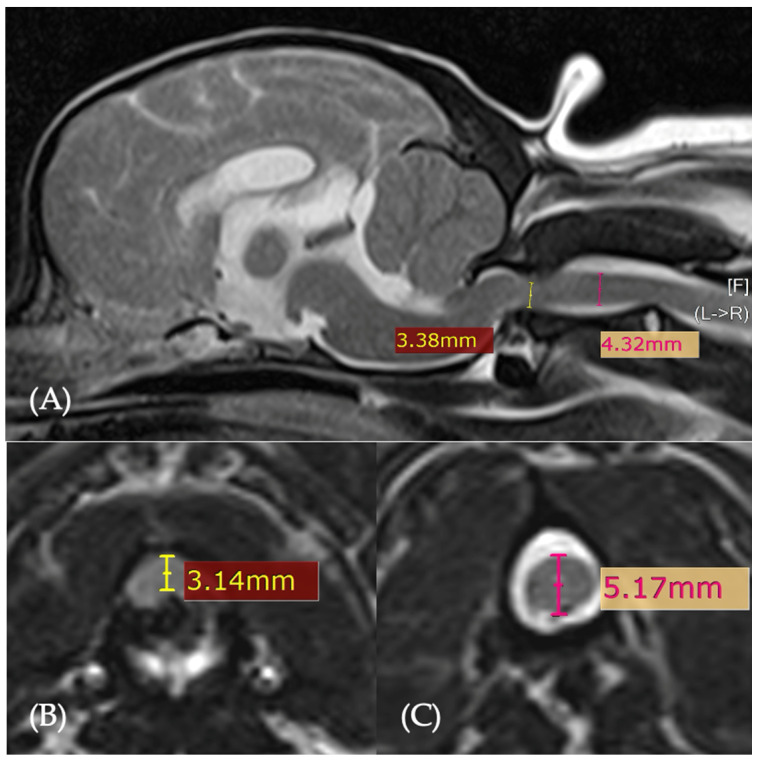
T2-weighted sagittal (**A**) and transverse (**B**,**C**) MR images. On the sagittal image (**A**), the dorso-ventral diameter of the spinal cord is measured at the level of the dens (yellow line) and at the mid-body of C2 (pink line). On the transverse images, (**B**) the cross-sectional area of the spinal cord is measured at the level of the dens (yellow line), and (**C**) at the mid-body of C2 (pink line) (Bray et al., 2023 [11]).

The percentage of spinal cord compression was calculated using sagittal and transverse images by comparing the spinal cord diameter at the level of the dens with the midpoint of the C2 vertebral body [11] (Figure 2).

##### Ventral Compression Index

**Figure 3:**
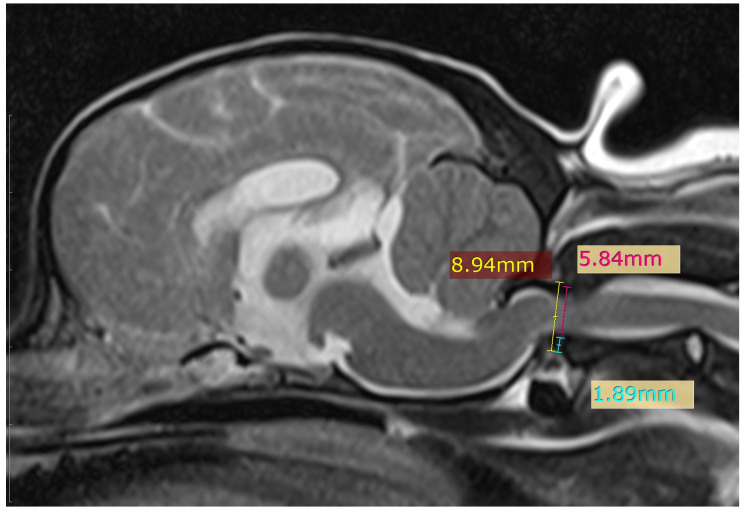
Measurement of the ventral compression index. The yellow line connects the midpoints of the ventral and dorsal arches of the atlas. The pink and blue lines represent the dorsal (DADI) and ventral (VADI) atlantodental intervals, respectively.

Ventral compression of the cervical spinal cord results from dorsal subluxation of the axis. Its value is derived from the ventral atlantodental interval (VADI) divided by the dorsal atlantodental interval (DADI) [14], as shown in Figure 3.

##### Syringomyelia

Syringomyelia refers to fluid penetrating the lining of the central canal, resulting in focal fluid collection within the spinal cord outside the central canal [23]. Using the British Veterinary Association/Kennel Club CM and SM Health Scheme [25], syringomyelia was graded as follows: grade 0 = normal; grade 1: central canal dilation (CCD) less than 2 mm in diameter; grade 2: syringomyelia (central canal dilation that has an internal diameter of 2 mm or greater), separate syrinx, or pre-syrinx with or without central canal dilation.

##### Chiari-Like Malformation

Anomalies in dogs similar to Chiari type 1 malformation in humans are characterized by a relatively reduced caudal fossa volume, resulting in overcrowding of the neural structures [23]. Using the British Veterinary Association/Kennel Club CM and SM Health Scheme [25], Chiari-like malformations are graded as follows: grade 0 = no Chiari malformation; grade 1: cerebellum indented (not rounded); grade 2: cerebellum impacted into, or herniated through, the opening at the rear of the skull (the foramen magnum).

##### Cerebellar Compression

**Figure 4:**
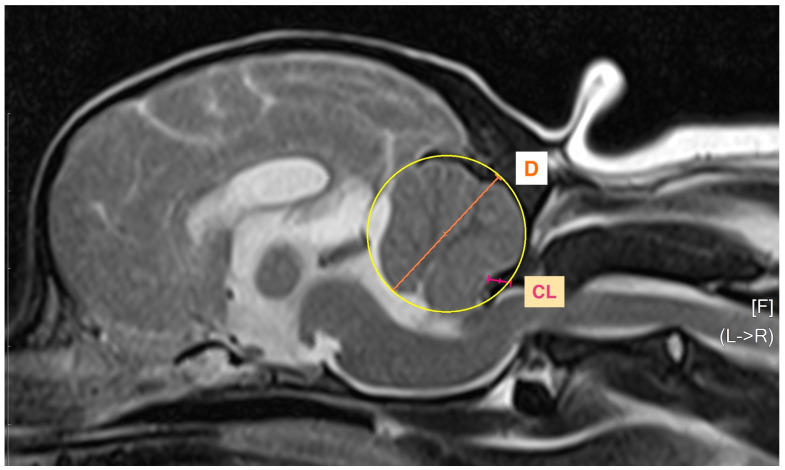
Measurement of cerebellar compression. The yellow circle represents the outline placed over the widest part of the cerebellum, from which the diameter (D, orange line) is measured. The orange line indicates the diameter of a circle over the widest part of the cerebellum, and the pink line indicates the distance from the outer margin of the subarachnoid space to the point of maximal neural compression.

Cerebellar compression is identified on MR images by evaluating the degree of displacement and crowding of the caudal cerebellum, which may be underestimated on other imaging modalities due to surrounding bone structures. According to Marino et al. [6], the CC index is calculated by dividing the distance from the outer margin of the subarachnoid space to the point of maximal neural compression (CL) by the diameter of a circle placed over the widest part of the cerebellum and multiplying it by 100 (Figure 4).

#### 2.2.3. Anesthesia and Surgical Technique

While preanesthetic medication varied according to the individual patient, the standard protocol consisted of intravenous diazepam (0.2 mg/kg), followed by an intravenous bolus of propofol (4–6 mg/kg) for induction. Anesthesia was maintained with isoflurane in oxygen and a continuous rate infusion (CRI) of morphine–lidocaine–ketamine (0.24 mg/kg/h, 50 µg/kg/min, and 0.6 mg/kg/h, respectively).

All surgeries were performed by a single board-certified neurosurgeon. Ventral stabilization was performed in all cases. Screws measuring 1.5–2.0 mm in diameter were placed with two screws inserted perpendicularly into the pedicle of the atlas. In the axis (C2), the cranial screw was oriented to achieve appropriate vertebral alignment. Nylon sutures were applied caudally to facilitate realignment. Polymethyl methacrylate (PMMA) was molded using a 5–10 mm circular syringe mold, which was removed after polymerization. Routine closure was performed in layers, including for the sternohyoid muscle, subcutaneous tissue, and skin. Additional intraoperative findings were recorded.

#### 2.2.4. Postoperative Management and Follow-Up

All dogs were admitted to the intensive care unit of Kasetsart University Veterinary Teaching Hospital at least one day after surgery. The postoperative management included opioid analgesia (duration based on pain score assessment) and infection prophylaxis. Dogs were discharged upon achieving a satisfactory physical examination. Follow-up neurological examinations were recorded and categorized into three time intervals: less than one week, one to four weeks, and four to ten weeks postoperatively.

### 2.3. Statistical Analysis

Statistical analyses were performed using IBM SPSS Statistics, Version 31.0 (IBM Corp., Armonk, NY, USA). Parametric or nonparametric tests were applied as appropriate. A paired *t*-test was used to compare the percentages of sagittal and transverse spinal cord compression. Associations were assessed using Spearman’s rank correlation and ordinal logistic regression. ROC curve analysis was used to evaluate the predictive ability of age, with the optimal cutoff determined using Youden’s index. A planned secondary analysis was performed to compare VCI values between survivors and non-survivors, including dogs excluded from the final outcome analysis due to perioperative death, using the Mann–Whitney U test. A two-tailed *p*-value < 0.05 was considered statistically significant.

## 3. Results

### 3.1. Animals

This retrospective study evaluated the association between preoperative magnetic resonance imaging (MRI) findings and surgical outcomes in dogs with atlantoaxial instability (AAI). Of the 28 dogs initially identified, eight were excluded (five due to postoperative deaths, two due to loss to follow-up, and one due to incomplete records), leaving 20 dogs in the final analysis.

The study population consisted predominantly of toy breeds, with Chihuahuas and Pomeranians representing the majority of cases (Table 1), consistent with the known breed predisposition for AAI. The median age was 2.8 years (range, 6 months–10 years), the median body weight was 2.11 kg (range, 1.3–4.3 kg), and there were 7 females and 13 males. Increasing age was significantly associated with poorer postoperative neurological outcomes at all evaluated time points (immediate postoperative period and at 1, 4, and 8 weeks). The receiver operating characteristic (ROC) curve analysis demonstrated good discriminatory ability of age for predicting postoperative neurological improvement (AUC = 0.843). The optimal cutoff value was 47.5 months, based on the highest Youden’s index (0.765), yielding a sensitivity of 100% and a specificity of 76.5%.

**Figure 5:**
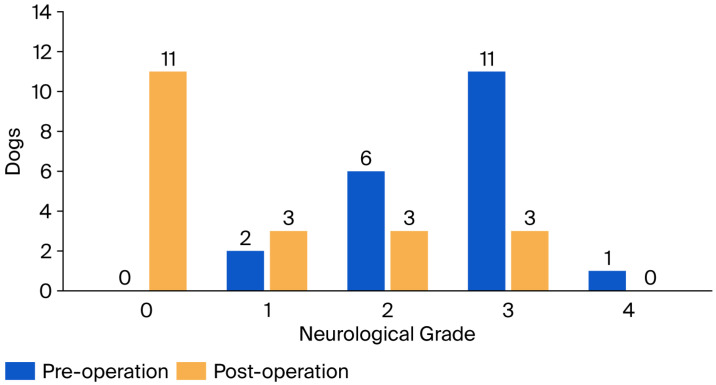
Distribution of neurological grades before and after surgical stabilization in 20 dogs with atlantoaxial instability (AAI).

Preoperatively, grade 3 was the most common neurological grade (*n* = 11), followed by grade 2 (*n* = 6), grade 1 (*n* = 2), and grade 4 (*n* = 1). Postoperatively, neurological grades were 0 (*n* = 11), 1 (*n* = 3), 2 (*n* = 3), and 3 (*n* = 3) (Figure 5) Spearman’s correlation revealed no significant association between preoperative neurological severity and the magnitude of improvement. However, ordinal logistic regression showed that dogs with severe preoperative grades (3–4) had significantly worse short-term outcomes (within 7 days and at 4 weeks) than those with mild grades (1–2).

### 3.2. MRI Findings

#### 3.2.1. Mechanical Spinal Cord Compression Parameters

Sixteen dogs were positioned with their head in extension (head position angle < 25°), whereas four dogs were positioned in flexion (angle ≥ 25°) during MRI acquisition. The mean ± standard deviation of the ventral compression index (VCI) was 0.58 ± 0.20 in the extended position and 0.40 ± 0.18 in the flexed position. No statistically significant difference in the VCI was observed between head positions (*p* = 0.138), suggesting that head positioning had minimal influence on VCI measurements in this cohort. The VCI did not appear to be associated with sex or breed in this sample. A moderate positive correlation was identified between VCI and preoperative neurological severity (Spearman’s ρ = 0.450, *p* = 0.047), indicating that increased ventral spinal cord compression was associated with more severe neurological deficits prior to surgical intervention. However, VCI was not significantly associated with postoperative neurological grade (Spearman’s ρ = 0.073, *p* = 0.759) or the magnitude of neurological improvement (Spearman’s ρ = 0.149, *p* = 0.529). These findings suggest that, although the ventral compression index (VCI) reflects the severity of preoperative spinal cord compression, it was not significantly associated with postoperative neurological recovery in the study cohort. Notably, dogs that died during the perioperative period—although excluded from the final outcome analysis—had significantly higher VCI values than survivors (Mann–Whitney U = 8.00, Z = −2.015, *p* = 0.044; exact *p* = 0.046).

The mean percentage of spinal cord compression was 37.58% for sagittal images and 35.36% for transverse images, with no significant difference between the two measurements (paired *t*-test: t = 1.066, df = 19, *p* = 0.300). No significant correlation was identified between sagittal spinal cord compression and change in neurological grade (Spearman’s ρ = 0.062, *p* = 0.794) or between transverse spinal cord compression and change in neurological grade (Spearman’s ρ = 0.135, *p* = 0.569). In contrast, preoperative sagittal spinal cord compression was significantly negatively correlated with neurological grade at 8 weeks postoperatively (Spearman’s ρ = −0.530, *p* = 0.017).

Atlantoaxial bands were identified through preoperative MRI in five dogs. Although dogs with dorsal spinal cord compression associated with atlantoaxial bands tended to have higher preoperative and postoperative neurological grades, these differences were not statistically significant (Mann–Whitney U = 20.0; exact *p* = 0.142 for preoperative grades; exact *p* = 0.266 for postoperative grades). Additionally, no significant difference in neurological improvement—defined as the change between preoperative and postoperative neurological grades—was observed between dogs with and without dorsal spinal cord compression/atlantoaxial bands (Mann–Whitney U = 31.0, exact *p* = 0.61).

#### 3.2.2. Ventricular Parameters

Lateral ventricular enlargement was identified in 15 dogs, with a mean ventricle-to-brain (V/B) ratio of 22.11 ± 5.2 and a mean ventricular index of 70.2 ± 9.2. Neither the ventricular-to-brain ratio nor the ventricular index was significantly associated with preoperative neurological severity (r = −0.125, *p* = 0.599; r = −0.403, *p* = 0.078, respectively), postoperative neurological status (r = 0.007, *p* = 0.977; r = −0.330, *p* = 0.156, respectively), or with the degree of neurological improvement.

In most cases, ventricular enlargement was symmetrical. Only one dog demonstrated moderate asymmetric ventriculomegaly, with an asymmetry ratio of 2.87:1.

#### 3.2.3. Craniocervical Structural Abnormalities

Eleven dogs presented with cerebellar compression. The Mann–Whitney U test demonstrated no significant difference in preoperative neurological grade between dogs with and without cerebellar compression (U = 34.50, Z = −1.27, *p* = 0.205). However, dogs with cerebellar compression exhibited significantly greater postoperative neurological improvement than those without compression (U = 21.00, Z = −2.24, *p* = 0.025).

While dogs without Chiari-like malformation tended to have slightly higher preoperative neurological grades, this difference was not statistically significant. In this cohort, the presence of Chiari-like malformation was not significantly associated with postoperative neurological status or neurological improvement.

#### 3.2.4. Intramedullary Pathology

Syringomyelia was identified in two dogs and was classified as SM1. While the presence of syringomyelia was not associated with preoperative neurological grades, postoperative neurological grades were higher in dogs with syringomyelia compared with dogs without syringomyelia (Mann–Whitney U = 1.0, exact *p* = 0.021). Fisher’s exact test demonstrated an association between grade change (improved vs. not improved) and syringomyelia status (*p* = 0.016).

## 4. Discussion

In the present study, increasing age was significantly associated with poorer postoperative neurological outcomes at all evaluated time points, including the immediate postoperative period and at 1, 4, and 8 weeks. Receiver operating characteristic (ROC) curve analysis demonstrated good discriminatory ability of age in predicting postoperative neurological improvement (AUC = 0.843), with an optimal cutoff value of 47.5 months. These findings suggest that older dogs are less likely to experience favorable neurological recovery following surgical stabilization. Previous studies in canine spinal cord injury have similarly reported that younger age is associated with improved surgical success and long-term outcomes, with some reports identifying ages below 24 months as a prognostic indicator [4,8]. The poorer outcomes observed in older dogs may be related to chronic spinal cord compression and its associated pathological changes. In chronic spinal cord injury, such as that associated with intervertebral disc extrusion (IVDE), progressive demyelination, white and gray matter loss, cyst formation, and extensive gliosis have been described. In severe cases, pan-myelomalacia with a complete loss of organotypic architecture may occur [26]. Although atlantoaxial instability (AAI) is generally regarded as a congenital condition, older dogs may have been exposed to prolonged or intermittent spinal cord compression before surgical stabilization. Chronic compression may result in cumulative intramedullary damage, reduced axonal integrity, and decreased potential for neurological recovery [26]. Therefore, the significant association between older age and poorer postoperative neurological outcomes observed in the present study may reflect the cumulative effects of chronic spinal cord pathology rather than age as an independent biological factor. This interpretation supports the importance of early surgical intervention before irreversible intramedullary damage occurs.

Emerging evidence suggests that cerebrospinal fluid (CSF) flow follows a bidirectional pattern coupled to the cardiac cycle. In addition, ventilation also contributes to CSF movement through changes in intrathoracic and intra-abdominal pressure [27]. When the spinal subarachnoid space is compressed, the systolic pressure wave creates a high-pressure compartment cranial to the obstruction and a lower-pressure compartment caudal to the obstruction. In cases of complete obstruction, the systolic pressure wave is transmitted through the spinal cord parenchyma caudal to the obstruction, and part of the wave is reflected cranially. This repeated transmission and reflection of pressure waves results in cyclic mechanical stress and the accumulation of extracellular fluid within the spinal cord [23]. Over time, this process may result in irreversible spinal cord damage. In the present study, we demonstrated that dogs with varying degrees of spinal cord compression secondary to atlantoaxial instability exhibited variable clinical manifestations, ranging from cervical pain to non-ambulatory tetraparesis. MRI-derived parameters of spinal cord compression—including the ventral compression index (VCI), the percentage of spinal cord compression, and the presence of atlantoaxial bands—were evaluated for clinical relevance. Although the underlying pathologies differ, similar observations have been reported in dogs with cervical and thoracolumbar intervertebral disc disease. Bach et al. [18] and Penning et al. [28] likewise found no significant association between preoperative neurological grade and the extent of spinal cord compression. Collectively, these findings indicate that neurological dysfunction may not be determined solely by the magnitude of static compression. Rather, in conditions such as atlantoaxial instability, spinal cord injury may reflect the combined effects of dynamic (concussive) and compressive forces in addition to secondary injury mechanisms. Additionally, our study found no correlation between MRI-derived spinal cord compression parameters and improvement in postoperative neurological grade. Another study investigating dogs with cervical disc extrusion reported no association between the degree of spinal cord compression and surgical outcome [18].

In the present study, no significant association was observed between preoperative neurological severity and the magnitude of long-term postoperative improvement. However, ordinal logistic regression revealed that dogs with severe preoperative grades (Grades 3–4) had significantly poorer short-term outcomes at 7 days and 4 weeks compared to those with mild grades (Grades 1–2). These findings are consistent with reports on cervical intervertebral disc extrusion, where ambulatory dogs achieved a faster initial recovery than non-ambulatory dogs, despite no significant difference in complete recovery rates by day 30 [18]. Furthermore, our results align with Takahashi et al. (2018) [29], who reported that preoperative neurological grades did not differ significantly between dogs with successful and unsuccessful clinical outcomes after ventral fixation for atlantoaxial instability. Taken together, these findings suggest that preoperative neurological severity influences the rate of recovery rather than the ultimate clinical outcome, as even severely affected dogs were capable of substantial improvement following surgical stabilization. From a pathophysiological standpoint, spinal cord injury reflects not only static compression but also secondary injury mechanisms. Mechanical trauma disrupts the blood–spinal cord barrier, leading to edema, impaired perfusion, inflammation, and the expansion of tissue damage. Axonal dysfunction may result from demyelination or supraspinal disconnection, while glial scar formation further limits regeneration [30]. Consequently, severe preoperative grades likely represent greater functional impairment related to contusion and secondary cascades rather than compression alone. Although decompression relieves mechanical pressure, neurological recovery depends on the resolution of these secondary processes [30,31], which may explain the slower early recovery observed in severely affected dogs.

Syringomyelia is thought to result from the obstruction or impairment of the normal pulsatile flow of CSF within the subarachnoid space [23]. In this retrospective study, only two dogs were diagnosed with grade 1 syringomyelia (SM). Nevertheless, dogs with syringomyelia had significantly higher postoperative neurological grades than dogs without syringomyelia, and dogs without neurological improvement had a higher prevalence of syringomyelia than those that showed clinical improvement. Furthermore, the presence of syringomyelia was associated with less neurological improvement following surgery. These findings should be interpreted with caution given the very small number of dogs with syringomyelia in this cohort (*n* = 2).

This study has several limitations. First, its retrospective design may have introduced selection bias and limited control over data completeness and follow-up consistency. Second, the relatively small sample size, particularly in subgroups such as dogs with syringomyelia, may have reduced statistical power and increased the risk of type II errors. Third, the follow-up duration was limited to short- and intermediate-term time points (up to 8 weeks), precluding an assessment of long-term neurological outcomes. Fourth, MRI measurements were obtained solely from preoperative imaging, and dynamic or postoperative changes were not evaluated. Additionally, head positioning during MRI acquisition may have influenced certain parameters, such as the compression index measurement. Future prospective studies with larger populations and standardized long-term follow-up are warranted to validate these findings. Fifth, this study did not systematically assess for atlantooccipital overlapping (AOO) or other craniocervical junction abnormalities beyond Chiari-like malformation, which may have influenced the results and is acknowledged as a study limitation.

## 5. Conclusions

In summary, increasing age and specific MRI findings—particularly severe ventral spinal cord compression and the presence of syringomyelia—were associated with less favorable outcomes following surgical stabilization for atlantoaxial instability (AAI). Although multiple MRI abnormalities were identified, not all were prognostically significant. These findings underscore the importance of comprehensive preoperative assessment and suggest that a younger age and the absence of syringomyelia may serve as favorable prognostic indicators in dogs undergoing surgical management of AAI.

This retrospective study was limited by a relatively small sample size, which may affect its statistical power and generalizability. Further prospective studies with larger populations and longer follow-up periods are warranted to better clarify the prognostic value of preoperative imaging findings and their association with long-term surgical outcomes.

## Figures and Tables

**Table 1:** Signalment of twenty dogs undergoing surgery for atlantoaxial instability.

Variable	Value (Median [Range] or *n* [%])
Age (years)	2.8 (6 months–10 years)
Body Weight (kg)	2.11 (1.3–4.3)
Gender	
Female	7
Male	13
Breed	
Chihuahua	11 (55%)
Pomeranian	7 (35%)
Shih Tzu	1 (5%)
Maltese	1 (5%)

## Data Availability

The data presented in this study are available within this article. The raw data supporting this study are available from the corresponding author upon reasonable request.

## Abbreviations

The following abbreviations are used in this manuscript:

AAI	Atlantoaxial instability
CCD	Central canal dilation
CC index	Cerebellar compression index
CSF	Cerebrospinal fluid
DADI	Dorsal atlantodental interval
MRI	Magnetic resonance imaging
SM	Syringomyelia
VADI	Ventral atlantodental interval
VCI	Ventral compression index
V/B ratio	Ventricle/brain ratio
